# Estimating risk of encapsulating peritoneal sclerosis accounting for the competing risk of death

**DOI:** 10.1093/ndt/gfz034

**Published:** 2019-02-28

**Authors:** Mark Lambie, Lucy Teece, David W Johnson, Michaela Petrie, Robert Mactier, Ivonne Solis-Trapala, John Belcher, Hilary L Bekker, Martin Wilkie, Ken Tupling, Louise Phillips-Darby, Simon J Davies

**Affiliations:** 1 Institute for Applied Clinical Sciences, Keele University, Staffordshire, UK; 2 Institute of Primary Care and Health Sciences, Keele University, Staffordshire, UK; 3 Department of Nephrology, Princess Alexandra Hospital, Centre for Kidney Disease Research, University of Queensland, Brisbane, Australia; 4 Renal Unit, Edinburgh Royal Infirmary, NHS Lothian, Edinburgh, Scotland, UK; 5 Renal Services, Glasgow Royal Infirmary, Glasgow, Scotland, UK; 6 Leeds Institute of Health Sciences, University of Leeds, Leeds, UK; 7 Renal Unit, Sheffield Teaching Hospitals NHS Foundation Trust, Sheffield, UK; 8 Kidney Patient Association, Sheffield Area Kidney Association, Northern General Hospital, Sheffield, UK

**Keywords:** age, encapsulating peritoneal sclerosis, peritoneal dialysis, peritoneal membrane, prognosis

## Abstract

**Background:**

Risk of encapsulating peritoneal sclerosis (EPS) is strongly associated with the duration of peritoneal dialysis (PD), such that patients who have been on PD for some time may consider elective transfer to haemodialysis to mitigate the risk of EPS. There is a need to determine this risk to better inform clinical decision making, but previous studies have not allowed for the competing risk of death.

**Methods:**

This study included new adult PD patients in Australia and New Zealand (ANZ; 1990–2010) or Scotland (2000–08) followed until 2012. Age, time on PD, primary renal disease, gender, data set and diabetic status were evaluated as predictors at the start of PD, then at 3 and 5 years after starting PD using flexible parametric competing risks models.

**Results:**

In 17 396 patients (16 162 ANZ, 1234 Scotland), EPS was observed in 99 (0.57%) patients, less frequently in ANZ patients (*n* = 65; 0.4%) than in Scottish patients (*n* = 34; 2.8%). The estimated risk of EPS was much lower when the competing risk of death was taken into account (1 Kaplan–Meier = 0.0126, cumulative incidence function = 0.0054). Strong predictors of EPS included age, primary renal disease and time on PD. The risk of EPS was reasonably discriminated at the start of PD (C-statistic = 0.74–0.79) and this improved at 3 and 5 years after starting PD (C-statistic = 0.81–0.92).

**Conclusions:**

EPS risk estimates are lower when calculated using competing risk of death analyses. A patient’s estimated risk of EPS is country-specific and can be predicted using age, primary renal disease and duration of PD.

## INTRODUCTION 

Encapsulating peritoneal sclerosis (EPS) is a serious, uncommon condition predominantly affecting patients exposed to peritoneal dialysis (PD). Due to the strong association between EPS and longer periods of exposure to PD treatment [1–3], an important management question for patients is when the risk of future EPS becomes sufficiently high to justify switching to haemodialysis (HD) to mitigate this risk. Appropriate decisions require accurate assessment of the risk of EPS, including an accurate estimation of the impact of exposure time of PD on EPS risk.

For some patients, even if the risk of EPS is thought to be high, it may still be relatively small in comparison to the risk of death. The challenge for safe and effective management is estimating when the risk of future EPS becomes sufficiently high to justify switching to HD, as there may be significant disruption to the patient’s quality of life and little length of life advantage. Appropriate clinical judgements and shared decisions with patients require an accurate risk assessment of EPS informed by a patient’s clinical indicators rather than population-based associations between incidence and exposure to PD treatment.

As death prevents the opportunity to develop EPS, death can be considered a competing risk to EPS. Analyses using methodology that does not appropriately incorporate competing events have been shown to overestimate the probability of occurrence of the event of interest [4–6]. Examples of competing risks in nephrology include time until peritonitis episode with competing events of technique failure/death/transplantation [7], end-stage kidney disease for diabetes patients with the competing event of death [8] and cause-specific mortality (cardiac death versus other causes of death) [9, 10].

When identifying risk factors in a competing risks situation, standard (cause-specific) survival methodology can be applied to estimate relative risks that reflect the direct association between the risk factor and a specific outcome. However, as the assumption of independent censoring can no longer be justified, competing risks methodology and the cumulative incidence function [11] are required to provide unbiased risk estimates. Competing risks analysis adjusts for the rate and risk of the competing events when estimating the absolute risk of the event of interest. Thus the risk of EPS for any given predictor variable (e.g. older age) would appreciably overestimate the true risk if that predictor variable was also associated with an increased risk of death and that competing risk was not taken into account.

To our knowledge, there has been no analysis of the incidence of EPS using a competing risks approach, such that previous risk estimates may have been misleadingly high. Furthermore, the prior identification of risk factors for EPS based on analyses that did not correctly account for the presence of competing risks may have been misleading. We sought to determine unbiased probabilities of EPS occurrence and death and consider the prognostic factors that enable identification of high-risk patients.

## MATERIALS AND METHODS

### Population

Data were extracted from two large registries, the Australia and New Zealand Dialysis and Transplant (ANZDATA) Registry and the Scottish Renal Registry (SRR). The ANZDATA data comprised all PD patients who started PD between January 1990 and December 2010 and were followed-up until October 2012. SRR data comprised PD patients who started PD between January 2000 and January 2008 with follow-up until April 2012 [10]. For both data sources, patients who started PD before they turned 18 years of age were excluded.

Follow-up started on the patient’s first date on PD and ended in the event of EPS, death or administrative censoring on October 2012 for ANZDATA and April 2012 for SRR. Follow-up continued whether or not a patient was still receiving PD, as EPS frequently occurs following PD cessation [12].

### Outcome and prognostic factors

In the ANZDATA registry, the EPS diagnosis was taken from comorbidity, cause of death or cause of PD cessation records as recorded by nephrologists from the contributing units, based on International Society for Peritoneal Dialysis (ISPD) criteria or, prior to that, those from Rigby and Hawley [1]. In the SRR, the EPS diagnosis was actively sought, with questionnaires sent to each centre and searching SRR data reported in hospital discharge statistics. Medical records were examined to ensure the ISPD criteria were met and excluded if another potential cause was present or if they lacked radiological or pathological confirmation.

Factors common to both data sets included age, gender, primary renal disease (as a surrogate for comorbidity as full information was not available in the SRR), diabetes status and cumulative exposure to PD in years. Primary renal disease was dichotomized as those considered to have a low risk of death (polycystic disease, isolated glomerulonephritis, chronic pyelonephritis) and those with a high risk of death (all others, including uncertain aetiology).

### Statistical methods

**Quantification of the risk of EPS**. Non-parametric estimates of the risk of EPS with cumulative time on PD were calculated using a Kaplan–Meier estimate and cumulative incidence function with death as a competing risk [13]. Direct comparisons of the estimates are presented.

**Prognostic factors for EPS**. Both univariable and multivariable models were fitted to investigate associations between the candidate prognostic factors and EPS. Flexible parametric models [14] using splines with three degrees of freedom to model the baseline log cumulative hazard were developed and both cause-specific hazard ratios (HRs) and subdistribution HRs estimated with a proportional hazards model. Non-linear associations were investigated using fractional polynomial terms and all first-order interactions tested for including registry with prognostic factors. Due to the disparity in prevalence of EPS, all analyses were stratified by registry. Proportional subdistribution hazards were assessed with time-dependent effects.

**Predictability of EPS**. The potential prognostic ability of a model predicting the risk of EPS was investigated through the development of prognostic models in PD patients at the start of treatment and 3 and 5 years after starting treatment. Multivariable flexible parametric models were used, assuming proportional subdistribution hazards, and stratified by registry, varying the baseline hazard functions between registries while retaining equal prognostic factor effects. The discrimination of the models was determined using Harrell’s C-statistic. The prognostic models were used to predict cumulative incidence function estimates for EPS and death separately over time for a high-risk and a low-risk patient group (age <40 years, low-risk primary renal disease versus age 60–80 years, high-risk primary renal disease) in patients still on PD.

All analyses used StataMP version 14 (StataCorp, College Station, TX, USA). The level of statistical significance was set at 0.05.

## RESULTS

### Characteristics and clinical outcomes

The analysis comprised 17 396 patients: 16 162 from the ANZDATA registry and 1234 patients from the SRR. Patient demographic characteristics (Table 1) illustrate systematic differences between the two populations; ANZDATA patients are older with a higher rate of diabetes mellitus.

**Table 1:** Patient demographics

Characteristic	ANZ				SRR			
	Total	EPS	Died	Censored	Total	EPS	Died	Censored
Patients, *n* (%)	16 162	65 (0.4)	8823 (54.6)	7274 (45.0)	1234	34 (2.8)	645 (52.3)	555 (45.0)
Age, median (IQR)	61.3 (49.3–70.7)	50.7 (40.1–57.7)	65.7 (55.7–73.9)	54.8 (42.4–65.7)	56.8 (42.9–67.8)	48.5 (39.6–60.7)	65.2 (55.2–72.1)	45.4 (35.6–57.0)
Male, *n* (%)	8887 (55.0)	31 (47.7)	4835 (54.8)	4021 (55.3)	675 (54.7)	19 (55.9)	364 (56.4)	292 (52.6)
Diabetes, *n* (%)	6602 (40.9)	16 (24.6)	4175 (47.3)	2411 (33.2)	329 (26.7)	7 (20.6)	232 (36.0)	90 (16.2)
High-risk primary renal disease, *n* (%)	10 238 (63.4)	26 (40.0)	6453 (73.1)	3759 (51.7)	740 (60.0)	16 (47.0)	479 (74.3)	245 (44.1)
Follow-up time, median (IQR), years	4.0 (2.2–6.8)	6.1 (3.8–8.0)	2.7 (1.4–4.7)	5.8 (3.7–9.4)	5.4 (2.5–7.8)	4.0 (3.5–5.8)	2.7 (1.2–4.5)	7.6 (6.1–9.6)
Years on PD, median (IQR)	1.8 (0.8–3.2)	5.5 (3.2–7.3)	1.9 (0.9–3.4)	1.6 (0.7–2.9)	1.7 (0.8–3.1)	4.0 (2.7–5.4)	1.4 (0.6–2.8)	1.9 (0.9–3.4)

IQR, interquartile range.

The most common outcome in both studies was death, occurring in 54.6% of ANZDATA patients and 52.3% of SRR patients. EPS was a rare event, with only 65 (0.4%) cases observed in the ANZDATA population and 34 (2.8%) in the SRR population. A total of 43.4% (ANZDATA) and 39.2% (SRR) remained on PD at 3 years, with 25.1% (ANZDATA) and 17.7% (SRR) at 5 years.

Comparing those patients who experienced EPS or death, patients who experienced EPS were significantly more likely to be younger and less likely to have either diabetes or a high-risk renal diagnosis (Table 1). There were also notably longer median follow-up times and median duration of PD treatment for those patients who developed EPS.

### Quantification of the risk of EPS

**FIGURE 1:**
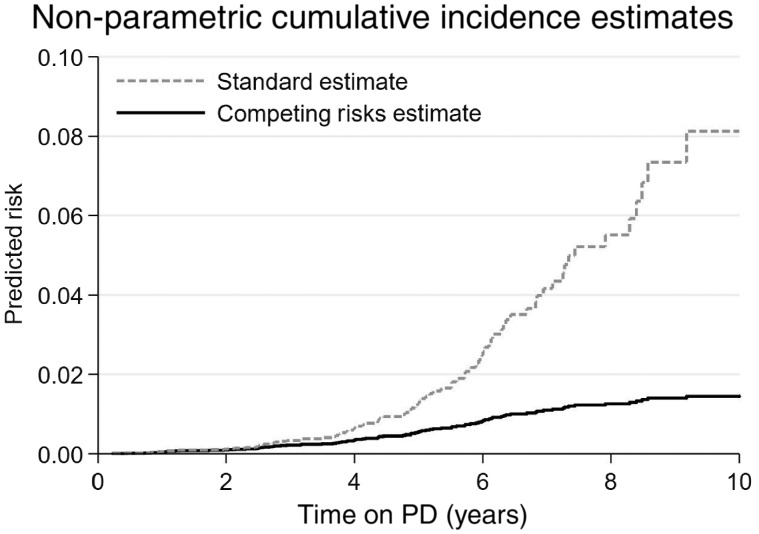
Risk of EPS calculated using ‘standard’ and competing risks approaches. The figure shows the non-parametric estimates of EPS risk over cumulative PD exposure. The standard approach, using the Kaplan–Meier estimate, is shown by the broken line. The competing risks approach, using the cumulative incidence function estimate, is shown by the solid line.

The difference in the risk of EPS according to whether competing risks were ignored (Kaplan–Meier) or accounted for (cumulative incidence function) are shown in Figure 1. The Kaplan–Meier curve derived markedly higher EPS risk estimates than those derived accounting for competing risks, particularly after 4 years of PD treatment.

### Prognostic factors for EPS

An increasing duration of PD was consistently associated with an increasing risk of EPS when using the duration of PD as the timescale, with a non-linear relationship suggested by graphing estimates from standard survival analysis and a linear one in the competing risks estimates (Figure 1). The same pattern was evident when the duration of PD was tested as a covariate in models using calendar time as the timescale (Table 2). In these models, age was not significantly associated with EPS risk in the standard survival analysis, but older age was associated with a lower EPS risk in the competing risks analysis {subdistribution HR 0.77 [95% confidence interval (CI) 0.68–0.88]}. Sex, diabetes and high-risk primary renal disease were not significantly associated with the risk of developing EPS. The effects of the prognostic factors were consistent across data sets, with no evidence of an interaction.

**Table 2:** Prognostic factors for EPS in patients starting PD

	Standard analysis				Competing risks analysis			
	Unadjusted		Adjusted		Unadjusted		Adjusted	
	HR (95% CI)	P-value	HR (95% CI)	P-value	SHR (95% CI)	P-value	SHR (95% CI)	P-value
Age (10 years)	0.932 (0.81–1.07)	0.324	0.899 (0.78–1.04)	0.146	0.724 (0.64–0.82)	<0.001	0.771 (0.68–0.88)	<0.001
Sex, male	0.896 (0.59–1.35)	0.602	1.024 (0.67–1.56)	0.911	0.871 (0.59–1.29)	0.491	1.171 (0.78–1.75)	0.442
Diabetes	0.935 (0.58–1.51)	0.784	1.319 (0.75–2.33)	0.338	0.500 (0.31–0.80)	0.004	0.854 (0.49–1.48)	0.574
High-risk PRD	0.770 (0.51–1.17)	0.223	0.707 (0.43–1.16)	0.170	0.465 (0.31–0.69)	<0.001	0.714 (0.44–1.15)	0.166
Duration of PD (per year)^a^	1.168 (1.11–1.23) 0.985 (0.98–0.99)	<0.001	1.172 (1.11–1.23) 0.984 (0.98–0.99)	<0.001	1.448 (1.35–1.55)	<0.001	1.415 (1.32–1.52)	<0.001

HR, cause-specific hazard ratio; PRD, primary renal disease; SHR, subdistribution hazard ratio.

a Fractional polynomial terms with powers 2 and 3 were selected to model the non-linearity of the duration of PD in the standard analysis.

### Predictability of EPS

Three competing risks prognostic models predicting the risk of EPS in PD patients at baseline and 3 and 5 years after the start of PD are presented (Table 3). The time on PD introduced an immortal time bias, so only age and primary renal disease were considered in the baseline model. The C-statistics demonstrated good discrimination for the baseline model in both the ANZDATA (0.74) and SRR (0.79) populations. Inclusion of the duration of PD exposure in the 3- and 5-year models improved the prognostic ability of the models further, with C-statistics between 0.81 and 0.92, but inclusion of whether a patient was currently on PD made no significant difference.

**Table 3:** Prognostic models for 5-year risk of EPS stratified by dataset

	Baseline		3 years		5 years	
Participants in cohort, *n* %	17 396 (100)		11 126 (64.0)		6975 (40.1)	
EPS, *n*	99		79		55	
	SHR (95% CI)		SHR (95% CI)		SHR (95% CI)	
Age (10 years)	0.756 (0.67–0.88)		0.739 (0.64–0.86)		0.733 (0.61–0.87)	
High-risk PRD	0.585 (0.39–0.88)		0.474 (0.29–0.76)		0.381 (0.21–0.71)	
Duration of PD (per year)	–		11.707 (4.36–31.43)		4.994 (2.78–8.98)	
	ANZ	SRR	ANZ	SRR	ANZ	SRR
Discrimination C-statistic (95% CI)	0.74 (0.65–0.82)	0.79 (0.70–0.88)	0.87 (0.81–0.94)	0.81 (0.75–0.88)	0.87 (0.80–0.94)	0.92 (0.89–0.96)

PRD, primary renal disease; SHR, subdistribution hazard ratio.

**FIGURE 2:**
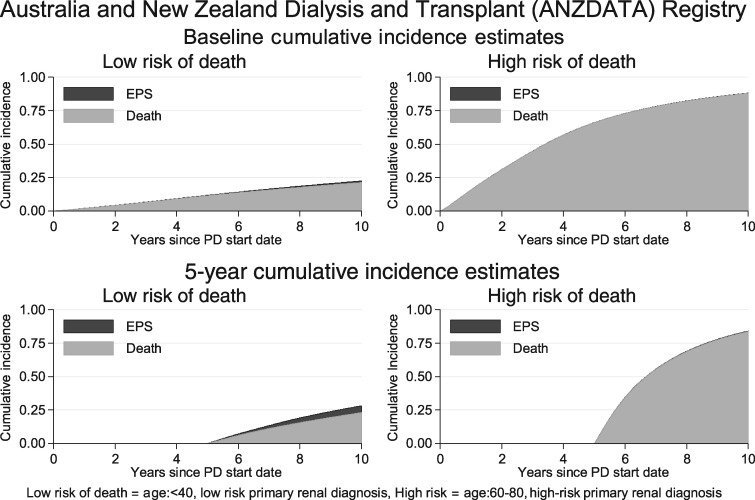
ANZDATA risks of EPS and death over time. Cumulative risk of EPS (black area) and death (grey area) over time in patient groups at low risk of death (left-hand column) and high risk of death (right-hand column) demonstrated for patients starting PD (top) and patients 5 years after starting PD (bottom).

**FIGURE 3:**
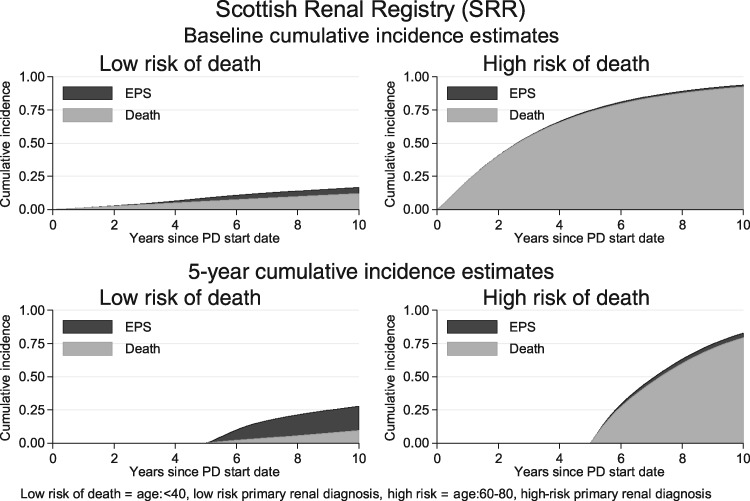
SSR risks of EPS and death over time. Cumulative risk of EPS (black area) and death (grey area) over time in patient groups at low risk of death (left-hand column) and high risk of death (right-hand column) demonstrated for patients starting PD (top) and patients 5 years after starting PD (bottom).

The potential utility of prognostic models for predicting individual EPS risk is demonstrated in Figures 2 and 3. Using the baseline risk of the ANZDATA population (Figure 2) for patients currently on PD, the probability of developing EPS was negligible both at the start of PD and 5 years after the start of PD for patients at high risk of death (ages 60–80 years, high-risk primary renal diagnosis). For the patients at low risk of death (age <40 years, low-risk primary renal diagnosis), the risk of EPS was larger. If the baseline risk of the SRR population was used (Figure 3), then the risk of EPS remained low compared with the risk of death in those patients at high risk of death, but became highly significant for patients at low risk of death 5 years after the start of PD. Plots for patients 3 years after the start of PD demonstrated intermediate risks between those in the graphs for patients at the start of PD and 5 years after the start (Supplementary data, Figure S1).

## DISCUSSION

We have demonstrated that estimation of EPS risk is strongly affected by death as a competing event and that statistical techniques need to take this into account. For example, neither age nor primary renal disease appeared to be directly associated with the rate of EPS occurrence, but they were significant predictors when death was incorporated as a competing event. Combining age, primary renal diagnosis (as a surrogate for co-morbidity) and the duration of PD accounted for the majority of the variability in EPS when allowing for death in the model.

Previous studies using models to estimate EPS risk have all used techniques such as the Kaplan–Meier estimator or the cumulative hazard [3, 10, 15, 16]. A fundamental assumption of these models is that EPS will eventually occur in all patients, a hypothetical scenario with no deaths. The effect is overestimation of the risk of EPS, known as competing risks bias [17]. The longer the follow-up and the greater the risk of competing events, the greater the overestimation. Estimates for EPS were strongly affected because, relative to the common competing event of death, EPS occurrence was rare.

Not accounting for the competing risks appropriately has likely led to the perception that the risk of EPS rises exponentially with the duration of PD [3, 15, 18]. We have clearly demonstrated that, when death is appropriately accounted for, this is not the case. This was illustrated both visually, with cumulative incidence function curves using the duration of PD as a timescale (Figure 1), and numerically, testing for a non-linear effect of PD duration in the survival models (Table 2).

One of the unexpected findings of this study was the magnitude of the difference in the risk of EPS between the ANZDATA and SRR data sets. Potential reasons include ascertainment bias, as the SRR data set identified EPS cases via questionnaires and follow-up phone calls to all centres. In comparison, the ANZDATA cases were identified through routinely returned registry codes, which may have been less reliable than specific study data. However, it may have in part also represented true differences in EPS risk between the different populations, possibly via differences in peritonitis, genetics and dialysate usage [19], as estimates of risk between countries have varied (e.g. between 0.6% and 6.6% for patients on PD for >5 years) and may vary within a country over time [20, 21]. Registry reports for some of the period included suggest a similar rate, but there are no data available on genotypes or dialysate usage. We overcame this disparity by stratifying the analysis to enable the underlying risk of EPS to differ between the registries while keeping the effect of the prognostic factors on the outcome stable.

The clinical impact of this analysis is that both the individual risk of death for a patient and the current level of EPS risk in the local PD population need to be considered when discussing whether to switch to HD to avoid EPS (illustrated in Figures 2 and 3). For all patients at high risk of death, the risk of EPS is low (right-hand column of both figures), even 5 years after the start of PD. The impact of different population risks of EPS becomes relevant in those patients at low risk of death, as illustrated by patients 5 years after the start of PD (bottom left plot, Figures 2 and 3). For this group in the ANZDATA population, the risk of EPS, while higher than in other groups, remained of debatable significance. For the corresponding group in the SRR population, the risk of EPS was greater than the risk of death, emphasizing the need for accurate national data collection to inform clinical risk prediction.

For patients at high risk of death/low risk of EPS, a switch to HD will take the patient off their preferred modality, with the attendant disruption to their quality of life, when the likely outcome is death whether or not the patient is switched. Furthermore, the risk of EPS spikes immediately after PD has stopped [22], so attempting to avoid EPS in this scenario could perversely increase the risk of EPS prior to death. For the small proportion of patients still on PD several years after starting with a low risk of death/high risk of EPS, and where transplantation is not imminent, a switch to HD may be appropriate, subject to an informed discussion with the patient.

A prognostic model that informed clinicians and patients of the absolute risks of EPS and death could help in the shared decision-making process. This was not possible due to the difference in baseline risk between the data sets, which would need to be defined for the population in which the prognostic model will be used. However, in the models developed, the duration of PD, age and primary renal disease were strongly predictive of EPS, demonstrating that a prognostic model could be successful, and the presence of competing risks bias demonstrates the need for a competing risks model to ensure accurate calibration. Previous studies have identified other risk factors that may help to identify patients at high risk of EPS, including impaired sodium sieving, poor ultrafiltration or decreased osmotic conductance to glucose, peritonitis episodes, dialysate inflammatory cytokine levels, exposure to non-biocompatible dialysis solutions and dialysate glucose exposure [10, 15, 22–26]. The predictive performance of the reported models indicates that adding further biomarkers to the prognostic model is unlikely to improve the prediction of EPS in a meaningful way, as a C-statistic of 0.92 (albeit on internal validation) is unlikely to improve dramatically for the overall population.

Limitations of this study include the fact that the model included a limited number of EPS events. There were some simplifications, such as not accounting for transplantation or switches to HD, which may have affected the risks of EPS and death differentially. This was mainly due to not having the appropriate information from the registry data sets. However, with more granular data, model extensions could involve multistate modelling to incorporate wait-listing for transplantation, having a transplant and being on HD as distinct states or joint modelling of cumulative time on PD. We were unable to include more predictors due to the limited prognostic factors common to both data sets and we were unable to determine a generalizable estimate of the baseline risk of EPS, as discussed previously. One other caveat is that there is no direct evidence that switching from PD to HD will mitigate the risk of EPS, although the strong association between duration of PD and EPS risk is likely to lead to this practice anyway.

In conclusion, the estimated risk of EPS can be substantially biased if death is treated as censored. When appropriately accounted for, the effect of duration of PD on EPS risk is linear, highly significant and, when combined with age and primary renal disease, provides accurate discrimination for EPS risk after prolonged periods of PD. There is a large apparent difference in risk between Scotland and ANZ, highlighting the need for robust data collection to ascertain the true risk in individual countries.

## FUNDING

This article presents independent research funded by the National Institute for Health Research (NIHR) Research for Patient Benefit (PB-PG-0610-22456). The views expressed are those of the authors and not necessarily those of the National Health Service, the NIHR or the Department of Health.

## AUTHORS’ CONTRIBUTIONS

S.J.D. conceived the study with input from J.B., D.W.J., L.P.D., M.W., K.T. and R.M. D.W.J., R.M., and M.P. acquired the data. L.T. performed the analysis with input from M.L. M.L. and L.T. co-wrote the manuscript. The results and the manuscript were reviewed by all co-authors.

## CONFLICT OF INTEREST STATEMENT

D.W.J. has previously received consultancy fees, research grants, speaker’s honoraria and travel sponsorships from Baxter Healthcare and Fresenius Medical Care. He is also the current recipient of a National Health and Medical Research Council Practitioner Fellowship. M.L. has previously received a consultancy fee from NxStage, speaker's honoraria from Baxter Healthcare and Fresenius Medical Care and research funding from Baxter Healthcare. S.J.D. has received research funding from Baxter Healthcare and speaker's honorarium from Fresenius Medical Care. J.B., L.P.D., L.T., H.L.B., R.M., M.P., I.S-T, M.W., and K.T. declare no conflicts of interest.

The results presented in this article have not been published previously in whole or part, except in abstract format.

## Supplementary Material

gfz034_Supplementary_Data
