# Supplementary material for: Estimating risk of encapsulating peritoneal sclerosis accounting for the competing risk of death
Source: Nephrol Dial Transplant. 2019 Feb 28;34(9):1585–91. doi: 10.1093/ndt/gfz034 (PMC6735880; doi:10.1093/ndt/gfz034)
Supplement: gfz034_Supplementary_Data [file gfz034_supplementary_data.docx]

Supplementary Figure 1: 5-year risk of EPS 3 years After PD Start

A - ANZDATA

B - SRR
